# Treatment Resistance Mechanisms of Malignant Glioma Tumor Stem Cells

**DOI:** 10.3390/cancers3010621

**Published:** 2011-02-10

**Authors:** Philip G.R. Schmalz, Michael J. Shen, John K. Park

**Affiliations:** 1 Surgical and Molecular Neuro-Oncology Unit, National Institute of Neurological Disorders and Stroke, National Institutes of Health, Bethesda, MD 20892, USA; E-Mails: philip.schmalz@nih.gov (P.G.R.S.); shenmic@nih.gov (M.J.S.); 2 Howard Hughes Medical Institute, National Institutes of Health Research Scholars Program, Bethesda, MD 20892, USA

**Keywords:** tumor stem cell, glioma stem cell, malignant glioma, glioblastoma, treatment resistance, chemotherapy, radiation therapy

## Abstract

Malignant gliomas are highly lethal because of their resistance to conventional treatments. Recent evidence suggests that a minor subpopulation of cells with stem cell properties reside within these tumors. These tumor stem cells are more resistant to radiation and chemotherapies than their counterpart differentiated tumor cells and may underlie the persistence and recurrence of tumors following treatment. The various mechanisms by which tumor stem cells avoid or repair the damaging effects of cancer therapies are discussed.

## 1. Introduction

Malignant gliomas are the most common primary intrinsic brain tumors of adulthood [1]. World Health Organization Grade IV malignant gliomas, also referred to as glioblastoma multiforme (GBM), account for the majority of malignant gliomas and carry a dismal prognosis [2]. Maximal safe surgical resection, radiation, and chemotherapy with temozolomide comprise the current standard of care. The more promising recently developed therapies include immunotherapies, receptor tyrosine kinase inhibitors, targeted toxins and the anti-angiogenic agent bevacizumab [3]. Despite these efforts, the median survival of patients with GBM has changed little in the past three decades and remains at approximately 15 months [4,5].

Over the past decade, there has been increasing recognition of a subpopulation of GBM tumor cells with stem cell characteristics. These glioma tumor stem cells (TSC) can be isolated from primary GBM specimens and propagated *in vitro* using serum-free culture conditions [6-10]. While most TSC express a combination of CD133, nestin, SOX2, LeX/SSEA-1, Bmi1, Ezh2, L1CAM and/or Olig2 [6,7,11-14], there is no single marker that is expressed both ubiquitously and exclusively in TSC. CD133 is a marker of normal neural stem cells [15]. Minority populations of CD133^+^ cells have been isolated from tissue derived from pediatric brain tumors and GBM specimens after expansion under stem cell conditions *in vitro* [6,9]. In a murine xenograft model, CD133^-^ cells showed engraftment without tumor formation while CD133^+^ cells demonstrated tumor formation with as few as 100 cells and have been termed “tumor initiating cells.” [16,17]. However, more recent work has shown that CD133^-^ cells can also fulfill the functional requirements for classification as TSC, generating neurospheres and expressing markers for all three neural lineages [18]. Thus, CD133 alone is not a complete marker for the TSC population. Nestin, an intermediate filament cytoskeletal protein, is a marker of normal neural stem cells in the developing CNS [11]. Neurosphere-forming cells isolated from primary GBM specimens express nestin, while non-sphere forming cells (non-TSC) lack nestin expression [9]. Thus, nestin serves as a nonspecific marker for neural stem cells and the TSC population, and forms an additional component of the TSC molecular phenotype. Sox2 is a transcription factor expressed by neural stem cells early in the developing neural tube [19]. Sox2 expression has been observed in a wide variety of brain tumor specimens [20]. Additionally, Sox2 expression has also been observed in undifferentiated neurospheres derived from GBM specimens [7]. However, Sox2 expression can also be demonstrated in whole GBM specimens, and though Sox2 has been described as a TSC marker, it is more likely an indicator of the aberrant differentiation that is characteristic of malignant gliomas [20]. In addition to the above phenotypic markers, Bmi1, Ezh2, L1CAM, and/or Olig2 are either overexpressed or correlated with CD133 expression in TSC, but are not sufficient to serve as unique characterization markers for these cells [6,7,20-25]. Because there is no single marker that is expressed both ubiquitously and exclusively in TSC, TSC are more rigorously defined functionally by their capacity for self-renewal, ability to form neurospheres *in vitro*, multi-lineage differentiation capability and greater tumorigenicity in immunocompromised mice when compared to matched differentiated tumor cells [10,12,16,25].

**Figure 1.**
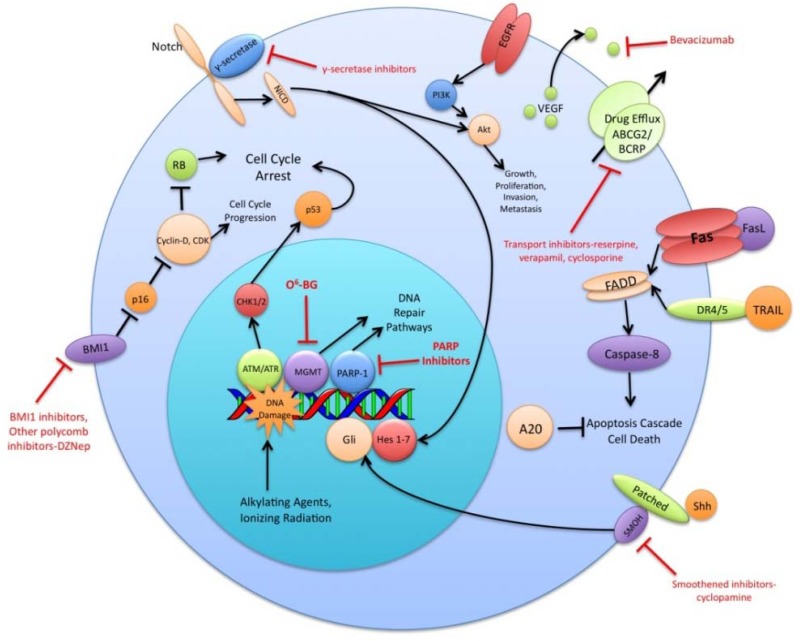
Mediators of TSC treatment resistance. Depicted are the various treatment resistance mechanisms and pathways differentially expressed or regulated in TSC versus their differentiated cell counterparts. Blocked red lines indicate ways to inhibit or block these mediators.

The preceding functional properties support the hypothesis that TSC are the cells responsible for the initiation and propagation of patient tumors. An extension of this hypothesis is that the overall treatment resistance of GBM tumors is due in large part to the treatment resistance of TSC in particular. Diverse mechanisms of radio- and chemo- therapeutic resistance have been demonstrated in the TSC population including increased expression of drug efflux transporters, a more robust DNA damage response, reduced sensitivity to apoptotic signals, increased expression of growth factors and dysregulation of transcription (Figure 1). This review will summarize recent research findings on these resistance mechanisms and discuss the various strategies used to circumvent these mechanisms.

## 2. Drug Efflux

ATP-binding cassette (ABC) proteins are a family of membrane transport proteins that use the energy derived from ATP hydrolysis to actively pump compounds out of the cytoplasm of cells. These transport proteins have a normal physiological role in the transport of bile salts in the gastrointestinal tract and are important components in maintaining isolated body compartments such as the blood-brain and blood-testis barriers [26]. ABC transport proteins often have nonspecific substrates, making them attractive candidates as mediators of multidrug resistance. An example of such a transporter is ABCG2 (also referred to as BCRP1), which has been demonstrated to confer resistance to multiple antineoplastic agents in a variety of cell types [27,28]. The expression of ABCG2 and P-glycoprotein (ABCB1) is also important for efflux of the Hoechst 33342 dye, a phenotypic characteristic that defines the “side population” of human tumor cells and stem cells [29-31].

CD133^+^ cells isolated from GBM tumors have increased ABCG2 expression and relative resistance to the antineoplastic agents temozolomide, carboplatin, etoposide, and paclitaxel [27]. Given the different mechanisms of action of each of these agents, a unifying explanation for the pan-resistance of these cells is the efflux of drugs via an ABC transport family protein. Long-term glioma cell lines cultured in serum-containing media also contain a minor side population of cells that express ABCG2 [30]. The switching of these cell lines to non-serum culture conditions promotes the growth of this ABCG2^+^ side population and increases the *in vivo* malignancy of the cell line. As a clinical correlate, it has been shown that GBM tumor samples obtained from patients in relapse are enriched in side population cells, suggesting that these cells are selected for by their resistance to chemotherapy [32].

A variety of ABC superfamily inhibitors have been tested on TSC, including clinically available compounds such as cyclosporine, reserpine, and verapamil [33]. These compounds, and ensuing generations of ABC superfamily inhibitors have been termed multi-drug resistance (MDR) reversal agents. Early efforts to use existing compounds clinically were met with limited success, as these compounds were often ineffective or toxic at doses required to inhibit ABC transporter function [34-36]. Subsequent generations of MDR reversal agents avoid the problems of primary toxicity that plagued early drugs. While primary toxicity is less of an obstacle, MDR reversal therapy is still troubled by the complex and hard to predict interactions between ABC transporters, their antineoplastic substrates, and MDR reversal inhibitors [37]. Many MDR reversal agents show nonspecific pharmacological activity. They can alter chemotherapy clearance, increasing plasma concentrations of chemotherapeutics beyond acceptable levels [38]. Also, the clinical efficacy of later generation compounds has been underwhelming [39,40]. Furthermore, in light of the fact that normal tissue stem cells express ABC transporters, as well as other multi-drug resistance genes, attempts to impact the tumor stem cell population via MDR reversal agents may damage the normal tissue stem cell population, counteracting potential increases in therapeutic index [41]. Alternative approaches to overcoming ABC transporter mediated resistance that have shown some promise *in vitro* include monoclonal antibodies and small peptides [42,43]. These and other more specific and potent inhibitors of this pathway may prove to be valuable components of a targeted chemotherapeutic regimen for malignant gliomas [26,37] (Figure 1).

## 3. Cell Cycle Control and DNA Repair

The cell cycle control system monitors and coordinates the progression of cells through the cell cycle. When functioning properly, new cells are produced when needed and defective cells, such as those with DNA mutations or damage, are either repaired or eliminated. An enhanced response, as well as a decreased response, to DNA damage, can confer a survival advantage to cells and lead to continued tumor growth despite treatment with radiation and chemotherapies that alter DNA.

ATM (ataxia telangiectasia mutated) and ATR (ataxia telangiectasia and Rad3 related) are among the first responders to genotoxic stress and DNA damage. They activate signal transduction pathways mediated by the effector kinases Chk1, Chk2, and Rad17. These effector or checkpoint kinases serve to activate p53 and inactivate cyclin-dependent kinases, thereby halting cell cycle progression and allowing for DNA repair [44-46]. *In vitro* and *in vivo* xenograft studies have demonstrated that CD133^+^ TSC are more resistant to ionizing radiation than matched CD133^-^ tumor cells [47]. The higher levels of phosphorylated, activated ATM, Rad17, Chk1 and Chk2 present in CD133^+^ cells are consistent with the enhanced capacity of these cells to sense and repair DNA damage. Upon inhibition of these checkpoint kinases, CD133^+^ cells are sensitized to ionizing radiation both *in vitro* and *in vivo* [47].

DNA alkylating agents, a commonly used class of drugs for many cancers, modify the guanines in DNA and lead to crosslinks, base pair mismatches, and enzyme-mediated fragmentation. Temozolomide (TMZ), the current first line chemotherapy for the treatment of malignant gliomas, methylates DNA at the *O^6^* and *N^7^* positions of guanine and the *N^3^* position of adenine [48]. Failure of the DNA mismatch repair system to find a complementary base for the methylated guanine leads to DNA nicking and eventual cell death. The repair enzyme *O^6^*-methylguanine DNA methyltransferase (MGMT) can, however, remove methyl groups from *O^6^*-guanine and allow the cell to continue replicating [49,50]. Expression profiling of CD133^+^ glioma TSC demonstrates a 32-fold increase in the level of MGMT transcripts relative to CD133^-^ tumor cells and indicates that this resistance mechanism is highly active in the glioma TSC population [32]. Synthetic substrates such as *O^6^*-methylguanine, *O^6^*-benzylguanine, and their chemical derivatives have been used in attempts to deplete MGMT in cells prior to initiating chemotherapy, but unacceptable myelotoxicity has been an obstacle [51,52] (Figure 1). Nevertheless, inhibition of MGMT, particularly in TSC, may one day prove to be a valuable component to a combination chemotherapy regimen.

## 4. Anti-Apoptotic Mechanisms

The extrinsic apoptotic pathway is activated by the specific binding of pro-apoptotic ligands to their cognate cell surface receptors. These ligands include FasL, which binds Fas, and TRAIL, which binds DR4/DR5 [53,54]. Following ligand/receptor binding, there is recruitment and clustering of the adaptor protein Fas-associated death domain (FADD) and procaspase 8 into a death-inducing signaling complex (DISC) [55-57]. Formation of the DISC brings the molecules into close proximity of one another and facilitates the catalytic processing of procaspase 8 into caspase 8 [58]. Caspase 8, in turn, is released into the cytoplasm where it activates the effector caspases 3, 6, and/or 7, eventually leading to apoptotic cell death [59].

Decreased sensitivity to apoptotic stresses has been shown to be a contributing factor to the increased resistance of TSC to ionizing radiation and alkylating chemotherapies. The differentiation of TSC with all-*trans*-retinoic acid increases apoptotic sensitivity, immature CD133^+^ U87 MG cells are more resistant to Fas-mediated apoptosis than their mature CD133^-^ counterparts and glioma TSC are poor responders to TRAIL-induced apoptosis [60-62]. One contributing mechanism to decreased apoptotic sensitivity may be the low or absent caspase-8 levels present in glioma TSC secondary to hypermethylation of the procaspase-8 gene promoter [63,64]. A strategy to facilitate TRAIL-induced apoptosis has been to enhance DR4/DR5 expression by inhibiting its degradation with bortezomib, a proteasome inhibitor [65].

L1CAM, a neural adhesion molecule central to cell growth, survival, migration, and axon formation, is differentially upregulated in the CD133^+^ fraction of gliomas [66,67]. *In vitro* treatment of CD133^+^ glioma cells with L1CAM-shRNA expressing lentiviruses causes reduced neurosphere formation and size and increased apoptosis while having little impact on CD133^-^ cells [23]. *In vivo* studies using L1CAM-shRNA have shown that mice bearing glioma xenografts with decreased L1CAM expression survived nearly twice as long as those bearing tumors with wild type L1CAM expression [23]. These studies demonstrate the potential clinical benefit of targeting L1CAM expression and function.

Another regulator of apoptosis is tumor necrosis factor-α-induced protein 3 (TNFAIP3). Also known as A20, it inhibits the NF-κB pathway and tumor necrosis factor signaling and confers tamoxifen resistance in breast cancer cells [68-71]. A20 is highly expressed in glioma TSC relative to non-stem GBM cells and siRNA knockdown of A20 in an *in vivo* murine xenograft model slows tumor progression [72,73]. In addition, *in silico* clinical analyses of tumor databases have indicated that a two-fold upregulation of A20 significantly correlated with decreased survival in patients with grade II or III, but not IV, astrocytomas [73,74]. Overcoming the relative apoptosis resistance of glioma TSC serves as a potentially attractive therapeutic strategy.

## 5. Growth Factors and Stem Cell Phenotype Maintenance Pathways

The vascular endothelial growth factors (VEGFs) are a family of cell surface or secreted proteins that bind and activate transmembrane receptors expressed primarily on the surface of endothelial cells. VEGF promotes endothelial cell proliferation, migration, and survival [75]. It has been shown that glioma TSC secrete markedly elevated levels of VEGF compared to matched non-TSC populations and hypoxia widens this difference [76]. *In vitro*, conditioned medium from TSC significantly increases endothelial cell migration and tube formation compared to that from non-TSC [76]. Bevacizumab, an anti-VEGF neutralizing antibody, specifically abolishes this effect. *In vivo*, it suppresses the growth of TSC-derived xenografts but has limited efficacy against non TSC-derived xenografts [76] (Figure 1). These data highlight the role of TSC in tumor angiogenesis and the importance of targeting this cell population when anti-angiogenic therapies are considered.

The Sonic Hedgehog (Shh)-Gli signaling pathway plays an important role in cellular proliferation and survival as well as in embryonic pattern formation [77,78]. The Shh glycoproteins act on a receptor complex consisting of two proteins, Patched and Smoothened. Upon binding of Shh, the inhibition of Smoothened by Patched is released. This results in activation of the downstream transcription factor Gli, which is integral for nervous system development [79,80]. In a panel of clinical brain tumor specimens, variable expression of Shh, Patched, and Gli1 was found, with Gli1 overexpressed on average in all tumors when compared to normal brain tissue [81]. This study also demonstrated that siRNA-mediated Gli knockdown results in decreased tumor cell proliferation and an increase in apoptosis. Several small molecule inhibitors of the Shh-Gli pathway have been investigated, including cyclopamine and SANT1. Treatment of glioma TSC with cyclopamine, an inhibitor of Smoothened, results in increased cell death, decreased neurosphere-forming ability and a reduction in the Hoechst dye-excluding side population of TSC [81,82] (Figure 1). Blockage of Hedgehog in TSC prior to injection into athymic mice causes loss of tumor-forming capacity [83]. In another study of patients with grade II to IV gliomas, the degree of expression of Shh, Patched, and Gli1 correlated with higher WHO grade and were independent predictors of poorer survival [84]. Shh pathway inhibitors may prove useful in their ability to specifically target and combat the glioma TSC population.

The Notch signaling pathway is critical for the specification of embryonic cell fate as well as the self-renewal of adult tissues [85-87]. In mammals, Notch receptor activation is mediated by the binding of one of two sets of transmembrane ligands, Delta-like ligands (Dll1-4) or Jagged ligands (Jag1 and Jag2). Ligand binding exposes the Notch receptor S2 domain to cleavage by a metalloprotease of the ADAM/TACE family [88]. Subsequent endocytosis of the transmembrane and intracellular domains of Notch allows a γ-secretase complex to further cleave Notch, releasing its active signaling portion, the Notch intracellular domain (NICD) [89-91]. The main cellular responses mediated by Notch are activation of transcription factors from the basic Helix-loop-helix family (bHLH) such as the Hairy Enhancer of Split genes (Hes1-7) that regulate nervous system and sensory organ development, somitogenesis and body patterning [92,93]. Notch signaling can either promote or suppress tumor development and growth in different cancer types [94,95].

Notch has been shown to promote the survival and proliferation of glioma TSC by maintaining them in an undifferentiated state. In one study, transfection of a TSC cell line with exogenous NICD resulted in a cell line with faster growth and improved colony and sphere-forming potential [96]. In another study, Notch activity was found to be elevated in CD133^+^ cells and inhibition of the Notch pathway with a γ-secretase inhibitor (GSI) resulted in decreased proliferative activity, a smaller CD133^+^ population, and decreased *in vivo* tumorigenicity [97]. The authors attribute these observations to the treated population displaying a larger fraction of differentiated cells due to GSI inhibition of the Notch pathway. Conversely, activation of Notch signaling increased the CD133^+^ cell fraction [97].

Notch has also been shown to play a critical role in regulating the radiation resistance of TSC. Inhibition of the Notch pathway with GSIs sensitizes glioma TSC to clinically relevant radiation doses, enhances their radiation-induced cell death and impairs their clonogenic survival but not the survival of non-stem glioma cells [98] (Figure 1). GSIs appear to act by reducing Akt activity and Mcl-1 levels rather than altering the radiation-induced DNA damage response of glioma TSC [98]. Additionally, expression of the constitutively active intracellular domains of Notch1 or Notch2 is protective while knockdown of Notch1 or Notch2 is sensitizing for glioma TSC exposed to radiation [98]. These studies indicate that modulation of the Notch pathway may be a promising strategy for overcoming the radiation resistance of glioma TSC.

Polycomb group proteins remodel chromatin and regulate important developmental genes by inducing transcriptional repression. Two members of the polycomb group family, BMI1 and EZH2, have been shown to be upregulated in CD133^+^ TSC. The BMI1 protein, originally studied as an oncogene in mantle cell lymphoma, is required for efficient self-renewal of hematopoietic stem cells as well as peripheral and central nervous system stem cells [99]. Cells rich in BMI1 and EZH2 associate with CD31^+^ endothelial cells, supporting the concept of a vascular niche for TSC [21]. *In vitro*, shRNA knockdown of BMI1 inhibits colony growth and leads to depletion of the CD133^+^ population, although growing the cells at a high density attenuates this effect. BMI1 has also been shown to be required for *in vivo* mouse xenograft tumor formation [21]. Pharmacologic inhibition of EZH2, an emerging therapeutic strategy, impairs both TSC self-renewal and speed of tumor formation [100] (Figure 1). Additionally, shRNA interference of EZH2 leads to poor TSC tumor formation *in vivo* [100]. Clinically, EZH2 expression increases with tumor grade and predicts poor prognosis [101]. These findings suggest that the BMI1 and EZH2 pathways may be promising avenues for the development of a combined targeted glioma therapy regimen.

## 6. Conclusions

Despite significant advances in our genetic and biochemical understanding of GBM pathogenesis over the past 30 years, there has been little improvement in patient survival. Numerous therapeutic strategies have been shown to be highly effective *in vitro* and in animal models, but have fallen short in the clinical setting. One explanation for this is that until recently, proposed therapies did not explicitly account for TSC. With the increasing appreciation of the role of TSC in tumor initiation and propagation, there has been a concomitant increase in studies exploring their role in overall tumor treatment resistance. Given this current body of evidence, it is clear that any preclinical treatments that appear to be effective on serum-cultured, differentiated tumor cells should also be tested on TSC. Another option would be to apply those therapies in combination with strategies that induce the differentiation of TSC. While there is much to be learned about TSC, the recognition of their treatment resistance mechanisms is a promising first step towards their eradication.
